# High Dose Dexmedetomidine: Effective as a Sole Agent Sedation for Children Undergoing MRI

**DOI:** 10.1155/2015/397372

**Published:** 2015-01-29

**Authors:** Sheikh Sohail Ahmed, Tamara Unland, James E. Slaven, Mara E. Nitu

**Affiliations:** ^1^Department of Pediatrics, Section of Pediatric Critical Care, Pediatric Sedation and Cardiovascular Intensive Care Unit, Riley Hospital for Children at Indiana University Health, Indiana University School of Medicine, 705 Riley Hospital Drive RI 4909 4B, Indianapolis, IN 46202, USA; ^2^Department of Pediatrics, Pediatric Procedural Sedation, Riley Hospital for Children at IU Health North, 11700 N. Meridian Street, Carmel, IN 46032, USA; ^3^Department of Biostatistics, Indiana University School of Medicine, 410 W. 10th Street, Suite 3000, Indianapolis, IN 46202, USA; ^4^Department of Pediatrics, Section of Pediatric Critical Care, Riley Hospital for Children at Indiana University Health, Indiana University School of Medicine, 705 Riley Hospital Drive RI 4909 4B, Indianapolis, IN 46202, USA

## Abstract

*Objective*. To determine the efficacy and safety of high dose dexmedetomidine as a sole sedative agent for MRI. We report our institution's experience. *Design*. A retrospective institutional review of dexmedetomidine usage for pediatric MRI over 5.5 years. Protocol included a dexmedetomidine bolus of 2 *μ*g/kg intravenously over ten minutes followed by 1 *μ*g/kg/hr infusion. 544 patients received high dose dexmedetomidine for MRI. A second bolus was used in 103 (18.9%) patients. 117 (21.5%) required additional medications. Efficacy, side effects, and use of additional medicines to complete the MRI were reviewed. Data was analyzed using Student's* t*-test, Fisher's exact test, and Analysis of Variance (ANOVA). *Main Results.* Dexmedetomidine infusion was associated with bradycardia (3.9%) and hypotension (18.4%). None of the patients required any intervention. Vital signs were not significantly different among the subgroup of patients receiving one or two boluses of dexmedetomidine or additional medications. Procedure time was significantly shorter in the group receiving only one dexmedetomidine bolus and increased with second bolus or additional medications (*P* < 0.0001). Discharge time was longer for children experiencing bradycardia (*P* = 0.0012). *Conclusion*. High dose Dexmedetomidine was effective in 78.5% of cases; 21.5% of patients required additional medications. Side effects occurred in approximately 25% of cases, resolving spontaneously.

## 1. Introduction

Sedation is often required in young children in order to obtain good diagnostic cross-sectional imaging like computed tomography scan (CT scan) and magnetic resonance imaging (MRI). Sometimes sedation is also needed for older children with complex neurodevelopmental disorders, such as autism and attention deficit hyperactivity disorder. Moderate sedation is unable to guarantee patient compliance; therefore, a deeper level of sedation is required [1, 2].

The success of sedation for MRI has typically been measured by two factors: the safety of sedation procedure (lack of adverse events) and its effectiveness (completion of diagnostic examination) [3].

It can be challenging to obtain the deep sedation level required to prevent the patient's movement while maintaining respiratory and hemodynamic stability. Also, limited access to the patient may pose a safety risk during MRI [4]. Therefore, it is very important to select the appropriate drugs and dosage to achieve those objectives [5].

Dexmedetomidine is a highly selective adrenoceptor agonist with a distribution half-life of six minutes and an elimination half-life of two hours [6]. Its success as a sedative agent varies depending on the dose and clinical situation [7]. Five prospective trials have evaluated the efficacy of dexmedetomidine for sedation during noninvasive radiologic imaging [8–12]. Dexmedetomidine can be associated with side effects of hypotension, bradycardia, and transient hypertension with loading dose. Dexmedetomidine has been used at our institution since 2006 for MRI and other noninvasive radiologic procedures. The purpose of this retrospective study is to present our institutional experience with dexmedetomidine in relation to efficacy and related side effects.

## 2. Materials and Methods

Collection of quality assurance data includes patient demographics, adverse events, physiologic variables, drug dosages, the time required to sedate the patient, time needed to obtain the imaging study, and recovery time. Data are entered into a database by two designated staff members. After approval by the Institutional Review Board, we conducted a retrospective analysis of all patients who received sedation for MRI from July 2007 to December 2012.

Institutional sedation policies are based on guidelines by the Joint Commission on Accreditation of Health Care Organization and American Academy of Pediatrics and were closely followed [13, 14]. Vital signs including pulse oximetry, heart rate, noninvasive blood pressure monitoring, and nasal capnography (with concomitant oxygen delivery via the nasal cannula) are continuously monitored and documented every five minutes throughout sedation.

At the start of sedation, intravenous dexmedetomidine was bolused at 2 *μ*g/kg over 10 minutes followed by a continuous infusion of 1 *μ*g/kg/h. Most of the patients were sedated by the end of the bolus; if not, a second bolus of 2 *μ*g/kg was repeated over another 10 minutes prior to the start of the maintenance infusion. Patients who continue to move after the second bolus or while the procedure is in progress, can compromise the quality of imaging and were given additional medications like midazolam or fentanyl as per physician discretion. The dose range used for Versed was 0.05 mg/kg–0.1 mg/kg to a max of 2 mg and for fentanyl was 0.5 mcg/kg–1 mcg/kg to a max of 50 mcg.

The goal was to achieve a minimum Ramsay Sedation Score (RSS) of 4 as assessed by a sedation nurse [15]. RSS is a clinically derived sedation score generally accepted as a tool for assessing the depth of sedation. Usually, a score of 4-5 is targeted to ensure appropriate sedation for diagnostic imaging studies [11, 16]. Peak onset of the sedation was defined as the time from the start of the loading dose to achievement of a Ramsay score of 4. Procedure time was the time of achieving the required Ramsay score to the end of the procedure (stoppage of drug administration). Discharge time was defined as time from the end of the procedure to actual time when the patient was discharged home [17]. Normal ranges for heart rates, blood pressure, and respiratory rates for data analysis were based on the published normal of Fleming et al. [17, 18].

A trained registered nurse provided continuous assessment and monitoring while the procedure was in progress under the direct supervision of a pediatric intensivist. Monitoring was continued until the patient was awake with a minimum Aldrete score of 9 points, and the patient has tolerated clear liquids prior to discharge [19, 20]. All the parents were provided with written discharge instructions and a direct phone number for further assistance or to report any adverse effects.

Database records were analyzed using dedicated statistical software SAS v9.3 (SAS Institute, Cary, NC). Data are expressed in terms of means and standard deviations. Changes in the vital signs associated with the use of dexmedetomidine from the baseline were evaluated and compared using Student's* t*-test and Mann-Whitney rank-sum test, depending on the distribution of the data. The entire study cohort was divided into three groups depending on dexmedetomidine bolus and additional medications received. Dexmedetomidine group A received one bolus; dexmedetomidine group B received two boluses; dexmedetomidine group C received one or two boluses and additional medications. Three groups were compared with respect to age, weight, blood pressure, heart rate, procedure time, and discharge time using Analysis of Variance. Incidences of bradycardia and hypotension were analyzed with Fisher's exact test, due to low cell counts. Discharge time of bradycardia and hypotensive patients was compared with normal cohorts using the* t*-test. A *P* value < 0.05 was considered statistically significant.

## 3. Results

544 patients were sedated using dexmedetomidine for MRI with 100% satisfactory completion. The most common indications included seizure disorder, developmental delay and behavioral disorder, autism, and neoplasia. Demographic characteristic and procedure and recovery times are presented in Tables 1 and 4. No significant group difference was found for gender, age, or weight.

**Figure 1:**
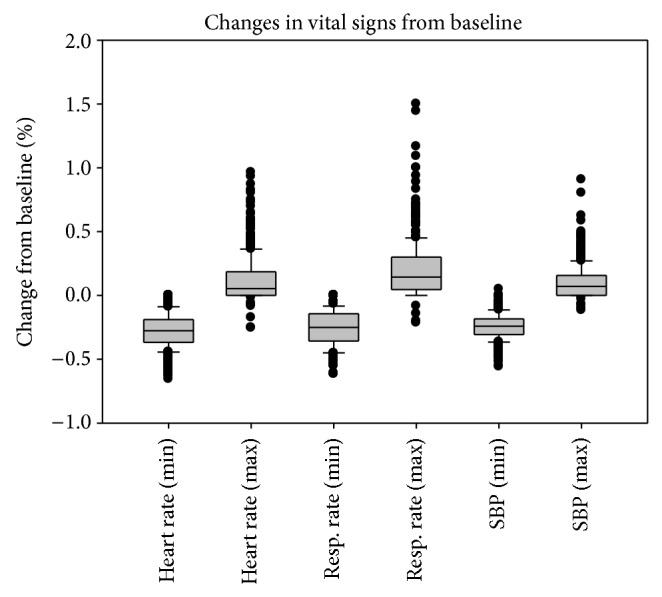
Minimal and maximal change in vital signs from baseline.

382 (70.2%) of the total patients received one-time bolus, while two boluses were given to 162 patients (29.8%). In the population of 544 patients, additional medications (fentanyl or midazolam) were required in 117 (21.5%) for spontaneous movements to avoid motion artifacts that can compromise MRI quality (54 patients with one-time bolus and 43 patients with two boluses). Dexmedetomidine-induced vital sign changes from baseline for the whole group are shown in Figure 1.

Hypotension (systolic blood pressure 20% below the normal limits) was observed in 100 patients (18.4%) and transient hypertension (systolic blood pressure >20% above age-specific high limits) was observed in 137 of the patients (25.2%). There were 210 (38.6%) patients who had a significant decrease in respiratory rate, that is, >20% from the baseline, but no desaturation, upper airway obstruction, or apnea events were observed and the decrease was minimal in 403 patients (74%). Most of the patients received prophylactic supplemental oxygen to maintain oxygen saturation >95% as per protocol, which makes it impossible to determine the actual incidence of desaturation at room air.

Patients were divided into three groups based on numbers of dexmedetomidine boluses and additional medications received. Comparisons of blood pressure, heart rate, and other clinical measures between these groups of patients are shown in Table 2. Procedure time was significantly shorter in dexmedetomidine group A (40.31 ± 19.40 min, *P* < 0.0001) compared to the other two groups. Oxygen, dose, maximum DBP, maximum respiration rate, and initial SPO2 had statistically significant (but clinically insignificant) differences between groups.

The occurrence of hypotension among all three groups did not reach a statistically significant value (*P* = 0.82). Decrease in blood pressure from the baseline was frequently observed; however, medical intervention was not needed to correct it. The incidence of hypotension was usually noticed to be towards the completion of procedure or after stopping the infusion, with a mean procedure time of 48.73 ± 25.46 minutes. Initial transient hypertension occurred in 137 patients approximately 20 minutes after the dexmedetomidine bolus and lasted for <15 minutes.

**Figure 2:**
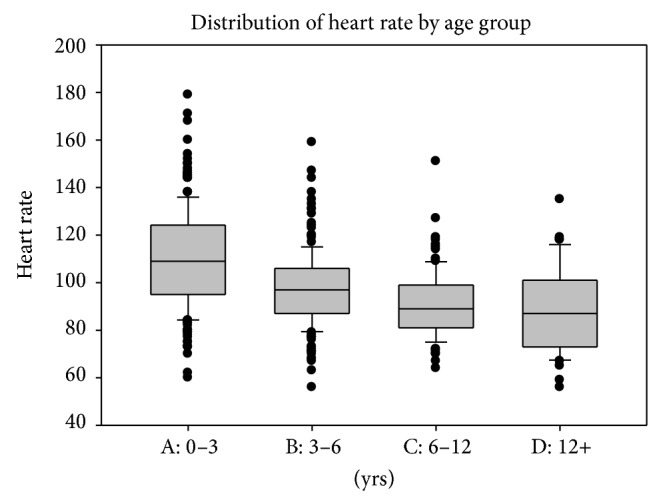
Heart rates for the 165 children below the age-specific normal range. Boxes represent normal range and tick marks denote heart rate values 20% below the normal range (15). Twenty-one children of the entire cohort of 544 (3.9%) were beyond the lower limit of normal by more than 20%.

In the population of 544 patients, 165 children (30.9%) had heart rates below the age-specific normal awake range during sedation (based on the published normal of Fleming et al.) [17]. Only in 21 children (3.9% of total cohort) did the lowest recorded heart rate fall >20% below the given baseline average range [12]. Bradycardia (HR < 60/min), as defined according to Pediatric Advance Life Support (PALS) guidelines, was observed in 10 children (4.5%) mostly in the age range of 1–3 years (Figure 2). All the patients with bradycardia were continuously monitored and assessed by the supervising intensivist for normal blood pressure and oxygen saturation of 95% or above. No patient required treatment. The incidence of bradycardia was high in patients in the age range of 1–3 years compared with the rest of the cohort. The precise etiology of the bradycardia observed in this analysis cannot be delineated as electrocardiogram monitoring is known to be significantly distorted by the magnetohydrodynamic (MHD) effect and is nondiagnostic within the bore of any MRI magnet.

Time to discharge when dexmedetomidine was used alone was 92 ± 25 versus 94 ± 37 min when additional medications were used (*P* = 0.46). Average discharge time among 165 children with bradycardia was longer and statistically significant compared to the other 372 children in the study population without bradycardia (99.08 ± 32.57 min versus 89.74 ± 25.54 min, *P* = 0.0012, Student's* t*-test). Gender was not associated with any change in discharge time (*P* = 0.30) (Table 3).

## 4. Discussion

Sedation is often necessary for obtaining quality MRI images in children. The ideal drug would allow optimal imaging, while maintaining hemodynamic and respiratory stability [21]. As reported in previous studies, inadequate sedation during MRI occurred in 5–15% of cases resulting in failure in 3.7%. This occurred more frequently in hyperactive, uncooperative, and older children [4, 22].

Dexmedetomidine, a selective alpha 2 adrenoceptor agonist, has a very safe therapeutic window with respect to respiratory depression. This quality offers a distinct advantage in procedural sedations, where the patient is not immediately accessible to the medical team. There are various studies demonstrating that dexmedetomidine is a good option for procedural sedation [10, 12]. Interestingly, the dose used varies greatly indicating an open debate regarding the best dosage.

Previous studies indicate that infusion of relatively low dose dexmedetomidine 0.1–0.7 *μ*/kg/h provides effective sedation [23–27]. In a study conducted on eight healthy volunteers receiving dexmedetomidine infusion of 0.2–0.6 *μ*/kg/h, the visual analog sedation scores and bispectral index scores dropped by 30–60% [23]. However, this level of sedation will likely not be conducive to pediatric MRI sedation, where higher doses of dexmedetomidine will be needed to accomplish the necessary sedation level. While dosing started low in initial reports, a recent one found that higher doses are required and have been well tolerated [12].

A variety of drugs have been used for MRI sedation. Pentobarbital is a short acting barbiturate and has been used frequently for MRI sedation. A comparison of pentobarbital and chloral hydrate was performed by Rooks et al. in 498 children for MRI sedation [28]. No significant and demonstrable differences were observed between the groups. Cardiovascular and respiratory depressions are the common side effects associated with pentobarbital. Several clinical trials have compared dexmedetomidine to propofol for pediatric procedural sedation. In a trial of 60 children undergoing MRI, patients were randomized to receive either dexmedetomidine or propofol. Adequate sedation was achieved in 83% and 90% of the patients, respectively. Onset, recovery, and discharge time were all significantly shorter in the propofol group. Adverse effects such as lower blood pressure and heart rate and respiratory rate were also found more in the propofol group [10]. Dexmedetomidine safety and efficacy for sedation during noninvasive radiologic procedures have been evaluated in four prospective trials [1, 8–10]. All of these studies reported that the use of high dose dexmedetomidine as a sole sedative resulted in high quality radiologic imaging with less use of additional rescue medicines. Our study also demonstrated that the use of high dose dexmedetomidine as sole agent is effective for MRI sedation. We used fentanyl and midazolam as rescue medicines in 117 (21.5%) of our patients to avoid imaging artifacts, suggesting that these agents make a significant contribution to the sedation strategy for a sizeable minority of patients.

In our study, the heart rate decreased significantly from baseline during sedation. This is an expected effect of anxiolysis and reflects higher baseline heart rate values from anxiety. Other studies reported a decrease in heart rate <20% from the baseline that was considered to be clinically insignificant in 86 (26%) patients [8]. To have a true comparison with previously published data, we use the same definition of bradycardia; that is, the lowest recorded heart rate during sedation fell >20% below the given baseline average range. Using that definition, the incidence of bradycardia in our study was 3.9% (21 children) in total cohort comparable to 4% as reported by Mason et al. [12, 29]. In 4.5% of the cases, the heart rate fell below 60 beats/min. This mostly occurred in children in the age range of 1–3 years. More importantly, during those periods of bradycardia, all patients maintained normal blood pressure and normal oxygen saturation (95% or higher).

Treating bradycardia in normotensive children with glycopyrrolate has been reported to be associated with hypertensive episode [29], which could be beneficial to children who are hypotensive and bradycardic at the same time. Similar to our findings, previous investigators have also reported the occurrence of transient hypertension with bolus administration of dexmedetomidine [30].

In our study, the occurrence of hypotension associated with high doses of dexmedetomidine was about 18%, observed immediately after stopping the infusion or towards the end of the procedure. None of the children needed any intervention, consistent with observation reported in other studies [12, 31].

Respiratory events make up a large proportion (5.5%) of sedation complications in children [2]. In some studies, rapid administration of large loading doses has been described to cause respiratory complications [32–34]. A loading dose of dexmedetomidine given over 2 minutes has been reported to cause irregular respiration, apnea, slight hypoxemia, and hypercapnia [35]. However, similar to ours, several other studies have reported trivial effect of dexmedetomidine on respiration [12, 34, 36] which is consistent with the notion that the risk of respiratory depression is minimal with careful dexmedetomidine sedation. Despite these supporting lines of evidence, monitoring of respiratory function during the administration of dexmedetomidine in those receiving midazolam or fentanyl, which may depress respiratory function, appears warranted [37].

Our reported discharge time of 92 minutes with dexmedetomidine only and 94 minutes when additional medicines were used is comparable to the 90 minutes' discharge time of Heard et al. [36]. Lubisch et al. observed a recovery time of 47 minutes [38] and Mason et al. [12] reported recovery times ranging from 24.8 minutes to 35.2 minutes, depending on the dose of dexmedetomidine used. In some studies, recovery time is the time lapsed until the patient meets the discharge criteria, while in our study, like few others, discharged time is defined as the actual time of leaving the recovery room to go home [36]. Discharge time of children experiencing bradycardia was longer than that for those who did not. In our study, 165 children with bradycardia had a mean discharge time which was statistically significant and longer than the other 372 children in the study without bradycardia (99.08 minutes versus 89.74 minutes; *P* = 0.0012). This probably could be due to the prolonged monitoring of these patients until the bradycardia resolved.

Recovery time previously reported after sedation with propofol for MRI has been 17 ± 8 minutes, almost half of the recovery time when dexmedetomidine was used as a sole agent [39]. In a second trial, forty children between the ages of 1 and 10 years were randomized to receive either a combination of midazolam + dexmedetomidine or propofol only for sedation during MRI. Recovery and discharge times were 15 minutes longer in the dexmedetomidine group. No adverse events were recorded in either group [40]. The fast recovery times of propofol must be weighed against the fact that it induced deeper sedation with significant hypotension and oxygen desaturation. Taking the results of these comparison trials as a whole, dexmedetomidine appears to provide a useful alternative to propofol for procedural sedation in children, with a longer time of recovery but a lower incidence of adverse effects. This is a good alternative to propofol for patients with soy and egg white allergy and also in institutions where use of propofol is strictly limited to be used by anesthesiologists.

The limitations of our study include its retrospective nature and single center experience. The current study presents a 100% success rate of sedation with dexmedetomidine for MRI. It could be asserted that the reported efficacy is due to the use of an intensivist based specialized sedation team rather than to dexmedetomidine itself. This is reasonably true to some extent as specialization and experience should increase both success and efficiency. In spite of that, this can be stated with confidence; much of the reported success is specifically a function of dexmedetomidine. This is a descriptive study and few, if any, conclusions can be drawn about safety because the occurrence of serious sedation related side effects is fortunately rare [41]. Additional prospective studies of the sedation for MRI in children using a greater number of patients are warranted to provide a true idea of safety.

In summary, high dose dexmedetomidine is an attractive and effective medication in children for MRI sedation. When using high dose dexmedetomidine as the only agent for pediatric MRI, it is not unusual to observe heart rate and blood pressure outside the established “awake” normal values. In our experience, these changes were pretty benign and were not associated with any adverse event. We conclude and recommend that, from hemodynamics and respiratory perspective, higher dose dexmedetomidine was well tolerated and is effective to use for successful completion of MRI, in the majority of pediatric patients.

## Figures and Tables

**Table 1:** Demographic characteristics (July 2007–December 2012).

	Dex A	Dex B	Dex C
Sex			
Male	180 (55.6)	70 (68.0)	65 (55.6)
Female	144 (44.4)	33 (32.0)	52 (44.4)
Age (months)	53.74 (38.49)	55.65 (33.39)	53.60 (42.47)
Weight (kg)	19.30 (10.43)	18.45 (8.78)	19.52 (13.73)

**Table 2:** Outcomes with DEX group—clinical measures.

	Dex A (*n* = 324)	Dex B (*n* = 103)	Dex C (*n* = 117)	*P* value
Oxygen liters/min	0.87 (0.48)	0.99 (0.56)	1.20 (0.61)	<0.0001^*^
Dose (mcg)	35.96 (17.37)	36.20 (10.38)	26.88 (17.58)	<0.0001^*^
DBP (initial)	66.28 (11.54)	67.91 (10.81)	65.29 (13.63)	0.2785
DBP (vital min)	39.75 (9.10)	39.52 (8.48)	40.34 (9.34)	0.8416
DBP (vital max)	80.29 (10.94)	81.38 (10.60)	84.32 (13.61)	0.0264^*^
SBP (initial)	112.50 (13.95)	111.93 (12.17)	113.10 (13.52)	0.8234
SBP (vital min)	84.61 (8.93)	83.60 (9.84)	84.91 (10.75)	0.5570
SBP (vital max)	122.66 (12.69)	122.17 (11.14)	124.78 (14.13)	0.2311
HR (initial)	99.86 (19.11)	103.16 (20.11)	101.40 (21.22)	0.3277
HR (vital min)	72.00 (13.94)	70.08 (12.49)	72.61 (14.50)	0.3559
HR (vital max)	110.69 (21.66)	111.12 (21.55)	114.97 (22.80)	0.1873
Resp. (initial)	20.45 (3.43)	20.36 (4.14)	21.24 (4.06)	0.1343
Resp. (vital min)	15.64 (4.10)	15.97 (5.42)	15.31 (4.24)	0.5413
Resp. (vital max)	24.27 (5.07)	25.84 (8.59)	26.25 (7.78)	0.00067
SPO2 (initial)	99.11 (1.14)	99.07 (1.12)	98.80 (1.40)	0.0548

Values are means (standard deviations); *P* values are from ANOVAs.

MTD: time to discharge, DBP min: lowest diastolic blood pressure, DBP max: highest diastolic blood pressure, SBP min: lowest systolic blood pressure, SBP max: highest systolic blood pressure, HR min: lowest heart rate, and HR max: highest heart rate.

^*^Significant association at *P* < 0.05.

**Table 3:** Discharge times for patients with and without bradycardia according to dexmedetomidine dosing protocol.

	Bradycardia	Mean (st. dev.)	No bradycardia	Mean (st. dev.)	*P* value
	*N*		*N*		
Dex A	97	96.23 (29.61)	224	89.83 (22.89)	0.0597
Dex B	31	90.35 (24.01)	70	94.04 (26.32)	0.5065
Dex C	37	113.90 (41.25)	78	85.63 (31.16)	0.0005^*^
All Dex	165	99.08 (32.57)	372	89.74 (25.54)	0.0012^*^

Values are means (standard deviations); *P* values come from Student's *t*-test.

^*^Significant association at *P* < 0.05.

**Table 4:** ASA (American Society of Anesthesia) classification and sedation times according to dexmedetomidine groups.

ASA^a^	Dex A	Dex B	Dex C
I	133 (41.1)	48 (46.6)	54 (46.6)
I/II	15 (4.6)	4 (3.9)	2 (1.7)
II	151 (46.6)	39 (37.9)	47 (40.5)
II/III	5 (1.5)	4 (3.9)	4 (3.5)
III	19 (5.9)	8 (7.8)	9 (7.8)
IV	1 (0.3)	0 (0)	0 (0)
Procedure time (minutes)^b^	40.31 (19.40)	46.42 (22.14)	57.04 (30.73)
MTD from end of test (minutes)	91.76 (25.23)	92.91 (25.57)	94.72 (37.00)

^a^American Society of Anesthesia' classification.

^b^Dex A procedure time significantly shorter than Dex B and Dex C.
